# The Mediating Role of Impulsivity in the Relationship Between Suicidal Behavior and Early Traumatic Experiences in Depressed Subjects

**DOI:** 10.3389/fpsyt.2020.538172

**Published:** 2020-11-10

**Authors:** Francesco Dal Santo, Juan José Carballo, Angela Velasco, Luis Jiménez-Treviño, Julia Rodríguez-Revuelta, Clara Martínez-Cao, Irene Caro-Cañizares, Lorena de la Fuente-Tomás, Isabel Menéndez-Miranda, Leticia González-Blanco, Mª Paz García-Portilla, Julio Bobes, Pilar A. Sáiz

**Affiliations:** ^1^Department of Psychiatry, University of Oviedo, Instituto de Investigación Sanitaria del Principado de Asturias, ISPA, Mental Health Services of Principado de Asturias, SESPA, Oviedo, Spain; ^2^Department of Child and Adolescent Psychiatry, Institute of Psychiatry and Mental Health, Hospital General Universitario Gregorio Marañón, Instituto de Investigación Sanitaria Gregorio Marañón, IISGM, Centro de Investigación Biomédica en Red de Salud Mental, CIBERSAM, Madrid, Spain; ^3^Department of Psychiatry, University of Oviedo, Centro de Investigación Biomédica en Red de Salud Mental, CIBERSAM, Instituto de Investigación Sanitaria del Principado de Asturias, ISPA, Oviedo, Spain; ^4^Department of Psychiatry, University of Oviedo, Centro de Investigación Biomédica en Red de Salud Mental, CIBERSAM, Instituto de Investigación Sanitaria del Principado de Asturias, ISPA, Mental Health Services of Principado de Asturias, SESPA, Oviedo, Spain; ^5^Department of Psychiatry, University of Oviedo, Oviedo, Spain; ^6^Department of Psychiatry, Fundación Jiménez Díaz Hospital, Universidad a Distancia de Madrid, Madrid, Spain; ^7^Instituto de Investigación Sanitaria del Principado de Asturias, ISPA, Mental Health Services of Principado de Asturias, SESPA, Oviedo, Spain

**Keywords:** suicidal behavior, stressful life events, childhood trauma, impulsivity, depression

## Abstract

**Background:** Depressed patients with early traumatic experiences may represent a clinically and biologically distinct subtype, with worse clinical outcomes and greater risk of suicide. Since early traumatic experiences alter development of systems that regulate the stress response, increasing sensitivity to stress and mood disorders later in life, certain personality features may influence coping strategies, putting individuals with depression and a history of early traumatic experiences at greater risk of suicidal behavior.

**Objective:** To determine whether impulsivity mediates the relationship between early traumatic experiences and suicidal behavior in patients with major depressive disorder (MDD).

**Methods:** The total sample consists of 190 patients [mean age (*SD*) = 53.71 (10.37); females: 66.3%], with current MDD (DSM-5 criteria). The Childhood Trauma Questionnaire-Short Form (CTQ-SF), the List of Threatening Experiences (LTE), and the Barratt Impulsiveness Scale-11 (BIS-11) were used to assess childhood and adulthood adverse life events and impulsivity, respectively. We developed mediation models by bootstrap sampling methods.

**Results:** Eighty-one (42.6%) patients had a history of previous suicide attempts (SA). CTQ-SF-Total and BIS-11-Total scores were significantly higher in MDD patients with previous SA. Correlation analyses revealed significant correlations between the CTQ-SF-Total and BIS-11-Total, CTQ-SF-Total and HDRS-Total, and BIS-11-Total and HDRS-Total scores. Regression models found that CTQ-SF-Total, BIS-11-Total, and HDRS-Total scores were associated with SA. Mediation analyses further revealed the association between CTQ-SF-Total and SA was mediated by the indirect effect of the BIS-11-Total score (*b* = 0.007, 95% CI = 0.001, 0.015), after statistically controlling for sex, the HDRS-Total, and the LTE-Total.

**Discussion:** Data suggest that impulsivity could mediate the influence of childhood trauma on suicidal behavior. This will help understand the role of risk factors in suicidal behavior and aid in the development of prevention interventions focused on modifiable mediators when risk factors are non-modifiable.

## Introduction

According to recent reports, depressed patients with early traumatic experiences may represent a clinically and biologically distinct subtype (1). Converging data from animal and human studies have illustrated how early-life adversity induces epigenetic regulation of multiple genes in the brain involved in the regulation of diverse biological processes (2), which may help understand the clinical heterogeneity found in this population. These neurobiological processes are of clinical importance given the association of early-life adversity with earlier age at onset of depressive illness, greater symptom severity, more comorbidity, poorer treatment response, and greater risk of suicide (1, 3).

Suicide prevention is a major public health concern. In 2017, it was estimated that, by the year 2020, about 800,000 people would die by suicide each year and at least 10–20 times more people would attempt suicide annually (4). Identifying those at greatest risk for completing or attempting suicide is a key aspect of suicide prevention (5). Psychiatric disorders, especially mood disorders, are among the most important risk factors for suicide (6). Reported prevalence of positive lifetime history of suicide attempts (SA) among depressed patients is high, although it varies among studies and settings. Figures from research conducted in outpatient and inpatient samples range between 30 and 40% in major depressive disorder (MDD) (7–9). Notably, mood disorder patients exposed to physical and/or sexual abuse during childhood are at greater risk of suicide vs. subjects without such experiences (10).

However, the specific mechanisms whereby adverse childhood experiences affect suicidality in adults with MDD remain to be elucidated. As not all children exposed to stressful life experiences develop psychopathology or suicidal behaviors, it is plausible to assume that there are additional variables that may help explain such an association. Since early traumatic experiences alter development of systems that regulate the stress response, increasing sensitivity to stress and mood disorders later in life (11), it is conceivable that increased lifetime stressors coupled with certain personality features would put individuals with depression and a history of early traumatic experiences at greater risk of suicide. For instance, some authors argue that is an accumulation of stressful life events over time, and not merely the presence of an isolated stressful life event, that relates to the occurrence of depressive symptomatology or suicidal behavior (12, 13). Conversely, with regard to personality features, it has been shown that poor emotional regulation strategies, poor emotional cognition, behavioral impulsivity, and self-criticism the increase risk of suicidal behaviors (14–16). More specifically, it has been proposed that childhood trauma may constitute an environmental risk factor for the development of impulsivity and SA in patients with depression (17), with data suggesting that childhood trauma could be related with problems in emotion regulation, which may in turn enhance impulsivity (18). However, the evidence suggests that impulsivity traits are part of a developmental cascade that increases suicide risk in a subset of suicide (not all suicides are associated with impulsive-aggressive behaviors) (19, 20), impulsivity being one of the most accepted endophenotypes when studying suicidal behavior (21).

The present study is aimed at exploring whether impulsivity traits could mediate the established relationship between early traumatic experiences and suicidal behavior in patients with MDD. We hypothesized that impulsivity would be related to childhood trauma and suicidal behavior in patients with MDD. We further expected that impulsivity could mediate the relationship between childhood trauma and suicidality in these patients. However, an alternative hypothesis in which depression severity could mediate such relationship will also be explored.

## Materials and Methods

### Participants

Cross-sectional study, including 190 Caucasian outpatients aged ≥18 years, recruited at the Mental Health Services in the area of Oviedo, Spain (population 331,936) from April 2016 to September 2018.

All participants had a diagnosis of current Major Depressive Disorder (MDD) according to the Diagnostic and Statistical Manual of Mental Disorders, Fifth Edition (DSM-5) (22). The exclusion criteria were comorbid psychiatric diagnoses other than tobacco use, Hamilton Depression Rating Scale score <15, intellectual disability, or any serious physical illness. Diagnoses were made by a psychiatrist and confirmed with the Structured Clinical Interview for DSM-5 (SCID-5) (23).

Suicide attempt (SA) was defined as a “self-initiated sequence of behaviors by an individual who, at the time of initiation, expected that the set of actions would lead to his or her own death” (22).

All participants received information about the purposes and protocol of the study and signed the informed consent before any study procedures were performed. The study was conducted in compliance with applicable laws and the provisions of the Declaration of Helsinki (24) and received institutional approval from the Clinical Research Ethics Committee of the Principality of Asturias.

### Assessments

All participants were assessed by well-trained interviewers using an “*ad-hoc*” protocol for sociodemographic and clinical data (sex, age, marital status, level of education, work status, tobacco use, antidepressant drug treatment, number of SA, and age at first attempt) (25). We employed the Spanish version of the 17-item Hamilton Depression Rating Scale (HDRS) (26, 27) to determine the severity of the depression. The Spanish version of the Columbia-Suicide Severity Rating Scale (28, 29) was used to assess the presence and characteristics of suicidal behaviors. Impulsiveness was assessed with the Spanish version of the Barratt Impulsiveness Scale-11 (BIS-11) (30, 31). BIS-11 is a 30-item scale that evaluates motor, attention, and planning components, which are consecutively characterized by action inhibition, decision-making, and planning understanding. Each item is rated on a scale of up to four points: 1 = rarely/never; 2 = occasionally; 3 = often; and 4 = almost always/always. The Childhood Trauma Questionnaire-Short Form (CTQ-SF) (32) is a 28-item retrospective self-report questionnaire to detect early stressors (emotional, physical, and sexual abuse, and emotional and physical neglect). This scale has been adapted for use in Spanish (33). Finally, the Spanish version of the List of Threatening Experiences (LTE) has been used to detect stressful life events (12 different stressors) in the 6 months prior to the evaluation (34, 35).

### Study Variables

We utilized four main variables during our analyses. These were: (1) History of previous SA (DSM-5 criteria); (2) Severity of childhood trauma measured with the CTQ-SF total score (CTQ-SF-Total); (3) Severity of impulsiveness assessed with the BIS-11 total score (BIS-11-Total), and (4) Severity of the depression assessed with the HDRS total score (HDRS-Total).

### Statistical Analysis

Statistical analyses were carried out using IBM SPSS Statistics for Windows, Version 24.0. The statistical significance was set at 0.05. Descriptive statistical analyses were expressed as means and standard deviations (*SD*) for quantitative variables, with frequencies and percentages used for categorical variables. Partial correlations were applied to explore the association between CTQ-SF-Total, BIS-11-Total, HDRS-Total, and LTE-Total, controlling for the following sociodemographic and clinical covariates: age, sex, level of education, work status, and recurrence of depressive episodes. Logistic regression models were built to explore the association of CTQ-SF-Total, BIS-11-Total, and HDRS-Total, with previous SA. Finally, two mediation models were developed to test the role of impulsiveness and depression in explaining the effect of early traumatic experiences on suicidal behavior. To control for their possible confounding effect, we included sex, age, and those variables with statistically significant differences between groups with or without previous SA in the bivariate analysis as covariates in the mediation models.

We ran the mediation models using model 4 (simple mediation) of the PROCESS macro for SPSS (v3.4) (36). The main goal of a simple mediation model is to test if the total effect of the independent variable (X) on the dependent (Y) can be partially or entirely by an indirect pathway (paths a and b) through a third variable, called mediator (M), located between X and Y. We used history of SA as the independent variable, CTQ-SF-Total as the dependent variable, and, alternatively, BIS-11-Total or HDRS-Total as mediators, performing a bootstrapped analysis with 5,000 replications to determine the significance of the mediatory effect. Bootstrapping is a data resampling technique from a non-parametric approach that allows hypothesis testing, estimating of size-effects, and constructing of confidence intervals without making any assumptions about the shape of the variable distribution or the sampling distribution of the statistic (37). Given the binary outcome of the dependent variable, we could not calculate the total effect of our model. This was because, in the case of a dichotomous Y, the regression coefficient for X in a model of Y without the mediator included is not equal to the sum of the direct and indirect effects of X (38).

## Results

### Sociodemographic and Clinical Characteristics

Table 1 shows the sociodemographic and clinical characteristics of the study sample. The final sample included 190 patients [mean age (*SD*) = 53.71 (10.37); females = 126 (66.3%)], of whom 81 (42.6%) had a history of previous SA. The mean number of SA was 2.78 (*SD* = 2.42). Ninety-seven patients (51.1%) had a single episode of MDD, while 93 patients (48.9%) had recurrent episodes, and the mean HDRS total score of the sample was 18.88 (*SD* = 5.77). Most participants had a primary education (*n* = 89, 46.8%), but only a minority (*n* = 35, 18.4%) were working at the time of the study. Compared with those without a previous SA, patients with a history of previous SA (*n* = 81, 42.6%) were significantly younger (51.11 vs. 55.69, *t* = 3.071, *p* = 0.002) and scored higher on the HDRS (20.02 vs. 18.03, *t* = −2.387, *p* = 0.018), CTQ-SF-Total (40.81 vs. 35.95, *t* = −2.450, *p* = 0.015), CTQ-Emotional Neglect (11.23 vs. 9.37, *t* = −2.431, *p* = 0.016), BIS-11-Total (67.21 vs. 62.22, *t* = −2.997, *p* = 0.003), BIS-11-Motor Impulsiveness (22.86 vs. 21.28, *t* = −2.066, *p* = 0.040), and BIS-11-Non-planning Impulsiveness (25.51 vs. 22.55, *t* = −3.729, *p* < 0.001). No differences were founds in the LTE score (2.98 vs. 2.69, *t* = −1.922, *p* = 0.056). All patients were on antidepressant drug treatment at the time of inclusion in the study.

**Table 1:** Sociodemographic and clinical characteristics of the sample (*n* = 190): [mean (*SD*)] or (*n*, %).

	**Total**	**No SA**	**Previous SA**	**Test (*p*)**
**Sociodemographic**				
Age (years)	53.71 (10.37)	55.69 (9.97)	51.11 (10.40)	*t* = 3.071, *p* = 0.002
Sex				χ^2^ = 2.690, *p* = 0.101
Female	126 (66.3%)	67 (61.5%)	59 (72.8%)	
Male	64 (33.7%)	42 (38.5%)	22 (27.2%)	
Level of education				χ^2^ = 4.565, *p* = 0.102
Primary	89 (46.8%)	54 (49.5%)	35 (43.2%)	
High School	74 (38.9%)	36 (33.0%)	38 (46.9%)	
University	27 (14.2%)	19 (17.4%)	8 (9.9%)	
Work status				χ^2^ = 1.538, *p* = 0.673
Active	35 (18.4%)	23 (21.1%)	12 (14.8%)	
Unemployed	60 (31.6%)	32 (29.4%)	28 (34.6%)	
Work incapacity	26 (13.7%)	14 (12.8%)	12 (13.7%)	
Retired	69 (36.3%)	40 (36.7%)	29 (35.8%)	
**Clinical**				
Recurrent depressive episodes	93 (48.9%)	51 (46.8%)	42 (51.9%)	χ^2^ = 0.477, *p* = 0.490
HDRS Total	18.88 (5.77)	18.03 (6.17)	20.02 (5.00)	*t* = −2.387, *p* = 0.018
LTE Total	2.81 (1.00)	2.69 (0.87)	2.98 (1.14)	*t* = −1.922, *p* = 0.056
CTQ-SF-Total	38.02 (13.24)	35.95 (11.49)	40.82 (14.90)	*t* = −2.450, *p* = 0.015
CTQ-SF-EA	7.75 (3.98)	7.26 (3.63)	8.42 (4.34)	*t* = −1.957, *p* = 0.052
CTQ-SF-PA	6.38 (2.63)	6.07 (2.15)	6.80 (3.12)	*t* = −1.805, *p* = 0.073
CTQ-SF-SA	5.89 (2.93)	5.63 (2.33)	6.23 (3.57)	*t* = −1.321, *p* = 0.189
CTQ-SF-EN	10.17 (5.17)	9.38 (4.75)	11.23 (5.53)	*t* = −2.431, *p* = 0.016
CTQ-SF-PN	7.83 (2.96)	7.61 (3.01)	8.12 (2.88)	*t* = −1.194, *p* = 0.234
BIS-11-Total	64.35 (11.59)	62.22 (11.11)	67.21 (11.67)	*t* = −2.997, *p* = 0.003
BIS-11-A	18.58 (4.12)	18.39 (4.19)	18.84 (4.04)	*t* = −0.735, *p* = 0.463
BIS-11-M	21.95 (5.29)	21.28 (5.37)	22.86 (5.06)	*t* = −2.066, *p* = 0.040
BIS-11-NP	23.81 (5.58)	22.55 (5.11)	25.51 (5.78)	*t* = −3.729, *p* < 0.001

*CTQ-SF, Childhood Trauma Questionnaire-Short Form; -EA, Emotional Abuse; -PA, Physical Abuse; -SA, Sexual Abuse; -EN, Emotional Neglect; -PN, Physical Neglect; BIS-11, Barratt Impulsiveness Scale; -A, Attentional Impulsiveness; -M, Motor Impulsiveness; -NP, Non-planning Impulsiveness; HDRS, Hamilton Depression Rating Scale; LTE, Brugha's List of Threatening Experiences; SA, Suicide Attempt*.

### Bivariate Correlations and Regression Analyses

Partial correlation analyses (Table 2) revealed a significant positive correlation between CTQ-SF-Total and BIS-11-Total (*r* = 0.253, *p* = 0.001), CTQ-SF-Total and HDRS-Total (*r* = 0.152, *p* = 0.040), and BIS-11-Total and HDRS-Total (*r* = 0.218, *p* = 0.003). No correlation was found between CTQ-SF-Total and LTE score (*r* = 0.035, *p* = 0.641), BIS-11-Total and LTE score (*r* = −0.033, *p* = 0.658), nor HDRS-Total and LTE score (*r* = 0.131, *p* = 0.075). Three logistic regression models were performed, including history of previous SA as a binary dependent variable and, alternatively, CTQ-SF-Total, BIS-11-Total or HDRS-Total as an independent variable. In the first model, CTQ-SF-Total was significantly associated with history of previous SA [β = 0.028, *p* = 0.014, odds ratio (OR) = 1.029 (95% Confidence Interval (CI) = 1.006 – 1.052)]. The second model indicated that there was a significant association between BIS-11-Total and history of previous SA [β = 0.039, *p* = 0.004, OR = 1.039 (95% CI = 1.012 – 1.067)]. The third model revealed a significant association between HDRS-Total and history of previous SA [β = 0.063, *p* = 0.020, OR = 1.065 (95% CI = 1.010 – 1.123)].

**Table 2:** Partial correlations between traumatic events, impulsiveness, and depressive symptoms [r (p)], after controlling for covariates (age, sex, level of education, work status, and recurrence of depressive episodes).

	**CTQ-SF-Total**	**BIS-11-Total**	**HDRS-Total**	**LTE-Total**
CTQ-SF-Total	-			
BIS-11-Total	0.253 (0.001)	-		
HDRS-Total	0.152 (0.040)	0.218 (0.003)	-	
LTE-Total	0.035 (0.641)	−0.033 (0.658)	0.131 (0.075)	-

*CTQ-SF, Childhood Trauma Questionnaire-Short Form; BIS-11, Barratt Impulsiveness Scale; HDRS, Hamilton Depression Rating Scale; LTE, Brugha's List of Threatening Experiences*.

### Mediation Analysis

**Figure 1:**
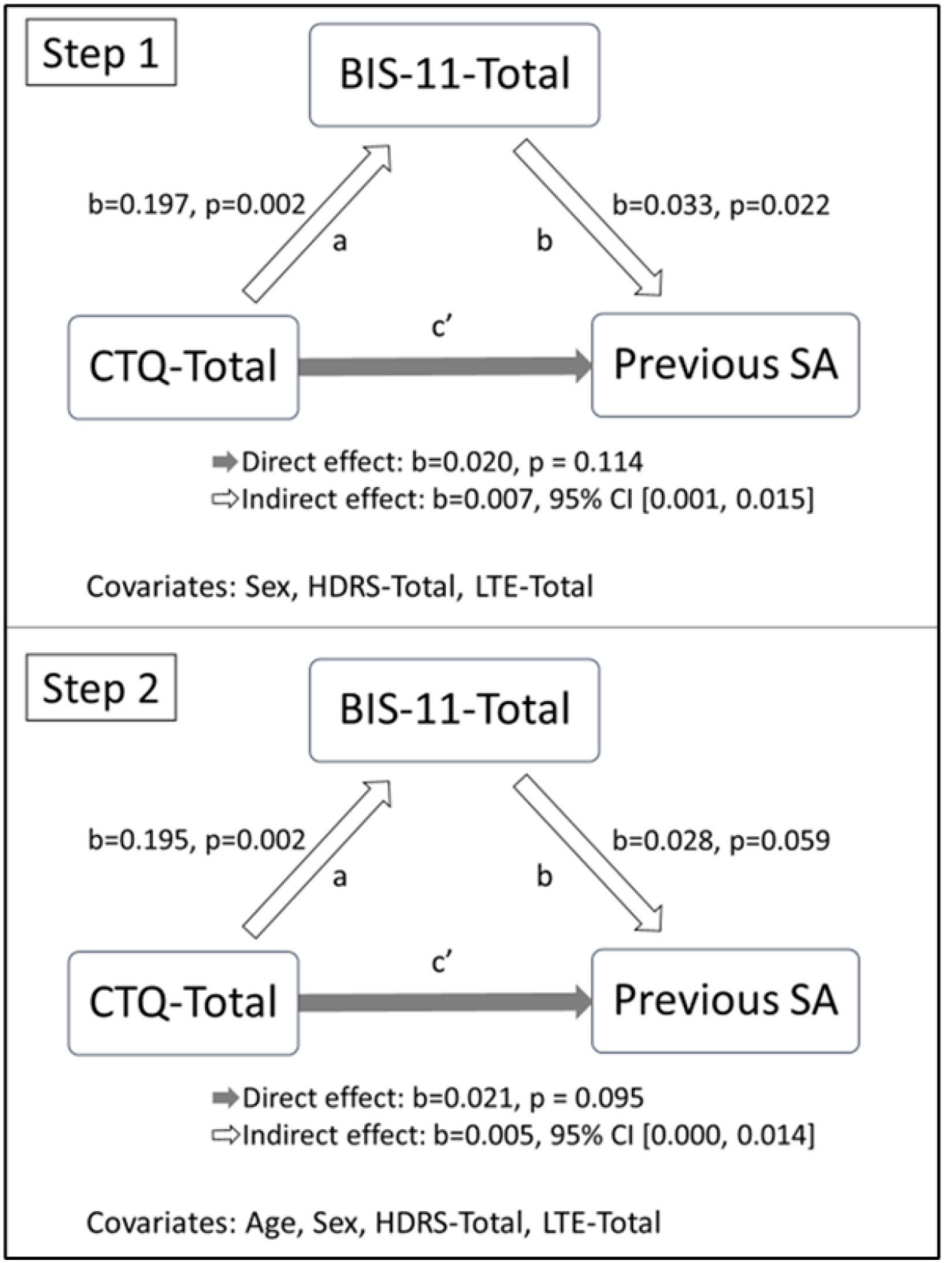
Mediation models of the association between the CTQ-SF-Total score and history of previous SA through BIS-11-Total score. CTQ-SF, Childhood Trauma Questionnaire-Short Form; BIS-11, Barratt Impulsiveness Scale; SA, Suicide Attempt; HDRS, Hamilton Depression Rating Scale; LTE, Brugha's List of Threatening Experiences.

The first mediation model was performed to test the role of impulsiveness in explaining the effect of early traumatic experiences on suicidal behavior. Age, sex, and the HDRS-Total (*p* = 0.018) were included as covariates in the model, along with the LTE-Total (*p* = 0.056). Due to the significant difference between the groups with or without history of previous SA concerning age, we conducted the analysis in two steps: (1) excluding the age from the list of covariates and (2) after its inclusion in the model. Figure 1 shows the mediation models illustrating the relationship between the dependent variable (CTQ-SF-Total), the mediator (BIS-11-Total), and the independent variable (history of SA). In the first step, after adjusting for the indirect effects of the mediator (BIS-11-Total), the direct effect (path c′) of CTQ-Total on history of previous SA was not significant (*b* = 0.020, *p* = 0.114). The bootstrapped 95% CI for the indirect effect of CTQ-Total on history of previous SA through BIS-11-Total (*b* = 0.007) was entirely beyond zero (0.001, 0.015), revealing that a mediation occurred. When age was added as a covariate, the indirect effect of CTQ-Total on history of previous SA through BIS-11-Total was no longer significant, as the bootstrapped 95% CI included the zero (0.000, 0.014).

The second mediation model was performed to test the role of the severity of the depression in explaining the effect of early traumatic experiences on suicidal behavior. Age, sex, and the BIS-11-Total (*p* = 0.003) were included as covariates in the model, along with the LTE-Total (*p* = 0.056). As for the former model, the analysis was conducted in two steps: (1) excluding the age from the list of covariates and (2) after its inclusion in the model. In the first step, we did not observe a significant indirect effect of CTQ-Total on history of previous SA through HDRS-Total (95% CI for the indirect effect: −0.001, 0.007). The second step of the analysis showed a similar non-significant result (95% CI for the indirect effect: −0.002, 0.006).

## Discussion

We conducted a cross-sectional study in order to evaluate the potential role of impulsivity traits and severity of depression as mediators of the established relationship between early traumatic experiences and suicidal behavior in depressed subjects (39). The main finding of our work was that impulsivity could mediate the influence of childhood trauma on suicidal behavior, after statistically controlling for sex, the HDRS-Total, and the LTE-Total. This association was no longer significant when age was entered as a covariate in the model. However, it should be noted that, probably due to the recruitment process, the group with previous SA included significantly younger patients, which may have influenced this discrepancy.

Previous research has illustrated how mental health problems, including suicidal behavior, may be the consequence of early traumatic events. Epigenetic mechanisms of gene regulation could clarify how early life experiences can leave permanent biological marks on the brain and can increase the risk of psychiatric conditions and suicide later in life (40). Levels of environmental adversity early in the developmental period affect stress-regulating pathways leading to long-lasting effects on stress responsivity during adulthood (41).

However, since not all depressed subjects who have experienced traumatic events in childhood display suicidal behavior in adulthood, some authors have posited that additional variables should be considered when attempting to clarify how early life experiences exert their influence over the life span. The stress sensitization theory proposes that early adversity and stress later in life are linked (42). It suggests that repeated stress early in life dysregulates stress response systems and lowers the threshold for reactivity and adaptive responses to subsequent stress. Similarly, the theory of stress proliferation, which posits that “stress begets stress,” helps explain how early traumatic experiences can result in secondary stressors (43). In the same vein, a chain reaction of adversity leading to accumulation of stressors throughout development has been reported (44, 45). Interestingly, in our sample, we do not find any correlation between stressors during childhood and adulthood, as assessed using CTQ and LTE scales, respectively. It is important to bear in mind that the LTE assesses stressful life events only in the 6 months prior to the sample evaluation. However, it may be that the direction of the association could vary if the presence of stressors during adulthood were explored over longer periods of adult life. On the other hand, the lack of association between stressors during adulthood and SA may be due to the fact that, in the present sample, the SA may be have occurred prior to the 6-months period assessed by the LTE.

Childhood adverse experiences challenge learning and academic achievement, compromising educational, workforce, and socioeconomic accomplishments in adulthood (46). Thus, the selection of negative social environments in adulthood may be not random, but rather the consequence of growing up in negative social environments (47). On the other hand, risky social environments mold coping styles, emotion regulation, and social cognition (39), and disturbances in these dimensions affect personality development. However, childhood adverse experiences have already been shown to increase impulsivity in suicidal patients (17, 48, 49). This is of clinical importance given that poor emotional regulation strategies, poor emotional cognition, behavioral impulsivity, and self-criticism are personality features that reportedly increase risk of suicidal behaviors (15, 16).

Our finding that impulsivity (as assessed by the BIS-11 scale) mediates the relationship between early traumatic experiences and suicidal behavior supports previous findings regarding the impact of recent stressors and certain personality features on suicidal behavior (6). However, at the same time, it adds to the previous literature a potential mechanism whereby adverse childhood experiences affect suicidality in adults with MDD.

However, a number of limitations of our work suggest that our results should be interpreted cautiously: the instruments used are based on patient reports, and no coefficient of agreement with interviewer diagnoses was computed. Furthermore, the information provided by subjects was not confirmed using other informants. The retrospective nature of the assessment was subject to recall bias and may have led to underreporting of early traumatic experiences; the clinical origin of the sample limits the generalizability of the findings, and, as analyses are cross-sectional, it is difficult to establish the real direction of the relationship found. However, childhood traumatic experiences and impulsivity are treated as single cumulative variables in this study, but they could alternatively be classified in different domains. Furthermore, the group with previous SA included significantly younger patients, which could be due to the recruitment process in our sample rather than to a causal association between younger age and SA, limiting the interpretation of the effect of age on the mediation models. On the other hand, pharmacological treatment has not been included as a covariate in the mediation analyses. Finally, there is a theoretical limitation regarding mediation models. As some authors have pointed out (50), mediation models are based on confirmatory analysis, and if data support the hypothesis, it does not mean that the hypothesis is true or correct, although it is plausible and probably useful. We posited a mediation relationship before we started the analyses, but we could not anticipate if it would be a partial or a total mediation relationship. However, based on the previous literature discussed above regarding childhood traumatic experiences, personality features, and suicidality, it is reasonable to think that the relationship between impulsivity and suicidal behavior is not spurious.

Despite these limitations, the results presented here have important implications. Our results suggest that the impact of childhood trauma on suicidal behavior in adulthood could be mediated by impulsiveness. This is important to better understand the role of risk factors in suicidal behavior, as well as for the development of prevention strategies that may focus on modifiable mediators when risk factors are non-modifiable. The presence of any of these mediators needs to be explored in depressed subjects, especially when there are known childhood adverse experiences. Alternatively, as “time does not heal all wounds” (51), it is of great importance from a public health standpoint not only to prevent childhood adversity but also to intervene once it occurs in order to avoid long-lasting negative mental health effects.

## Data Availability Statement

The datasets generated for this study are available on request to the corresponding author.

## Ethics Statement

The studies involving human participants were reviewed and approved by Clinical Reseach Ethics Committee of the Principality of Asturias. The patients/participants provided their written informed consent to participate in this study.

## Author Contributions

FD, JC, LJ-T, IC-C, and PS analyzed and interpreted the data and wrote the manuscript. AV, JR-R, CM-C, LF-T, IM-M, LG-B, M^a^G-P, JB, and PS followed up patients and performed/assisted in evaluations. FD, JC, M^a^G-P, JB, and PS designed the study, analyzed data, contributed to data interpretation, and wrote the manuscript. All authors read and approved the final manuscript.

## Conflict of Interest

The authors declare that the research was conducted in the absence of any commercial or financial relationships that could be construed as a potential conflict of interest.
